# A Rare Presentation of Sarcoidosis: Dysphagia, Pancytopenia, and Acute Renal Failure

**DOI:** 10.7759/cureus.25600

**Published:** 2022-06-02

**Authors:** Deepesh Yadav, Sailendra Shah, Ghassan Bachuwa

**Affiliations:** 1 Internal Medicine, Hurley Medical Center, Michigan State University College of Human Medicine, Flint, USA

**Keywords:** bronchoalveolar lavage (bal), acid-fast bacilli (afb), esophagogastroduodenoscopy (egd), fine needle aspiration cytology (fnac), intravenous immunoglobulin (ivig)

## Abstract

Sarcoidosis is a multisystem inflammatory chronic disorder that can virtually affect any organ system in the body. Most commonly affected organs are the intrathoracic structures with 90% of the reported cases involving the lungs. Esophageal involvement in sarcoidosis is extremely rare. Involvement of the esophagus and kidney along with hematological involvement is extremely uncommon in the same patient. Here, we present a case of a 58-year-old gentleman with a similar rare presentation. The patient presented with shortness of breath, productive cough, fatigue, and difficulty in swallowing, along with a weight loss of 20-30 pounds over three months. Laboratory workup was significant for leukopenia (2900 K/UL), serum creatinine level of 2.7 mg/dL (baseline: 1.2-1.7), and raised angiotensin-converting enzyme level at 187 nmol/ml/min. Chest X-ray showed bilateral widespread fine reticulonodular opacities, chest CT showed extensive bilateral reticulonodular opacities throughout the lung parenchyma, and fine-needle aspiration cytology of the right lung showed noncaseating granulomas. No fungal or acid-fast organisms were identified, and no evidence of malignancy was seen. Special stains for fungal (Grocott's methenamine silver and periodic acid-Schiff) and acid-fast organisms (acid-fast bacilli (AFB) and fluorescent AFB) were negative. Esophagogastroduodenoscopy (EGD) with gastric biopsy showed acute and chronic inflammation and no intestinal metaplasia, dysplasia, or malignancy was identified. Bronchoalveolar lavage was done, which showed macrophages (74%), neutrophils (6%), eosinophils (3%), and lymphocytes (17%), and was negative for malignant cells. QuantiFERON and AFB sputum/*Mycobacterium tuberculosis* polymerase chain reaction were negative. The patient was initially started on intravenous fluids and calcitonin, which significantly improved renal function and the calcium status of the body. Then prednisone 40 mg daily was started, which improved swallowing and breathing. After a week, prednisone was changed to 20 mg daily and was continued at the time of discharge.

## Introduction

Sarcoidosis is a noncaseating granulomatous and multisystemic disease of unknown etiology. The classical presentation of sarcoidosis includes systemic symptoms of fatigue, weight loss, and night sweats along with pulmonary changes such as hilar adenopathy seen in imaging. However, a significant proportion of patients with sarcoidosis are incidentally diagnosed and a wide variety of symptoms can be involved concomitantly. Noncaseating granulomas are the defining pathologic findings in sarcoidosis, and a biopsy is required for confirmation of diagnosis [1,2].

## Case presentation

**Figure 1:**
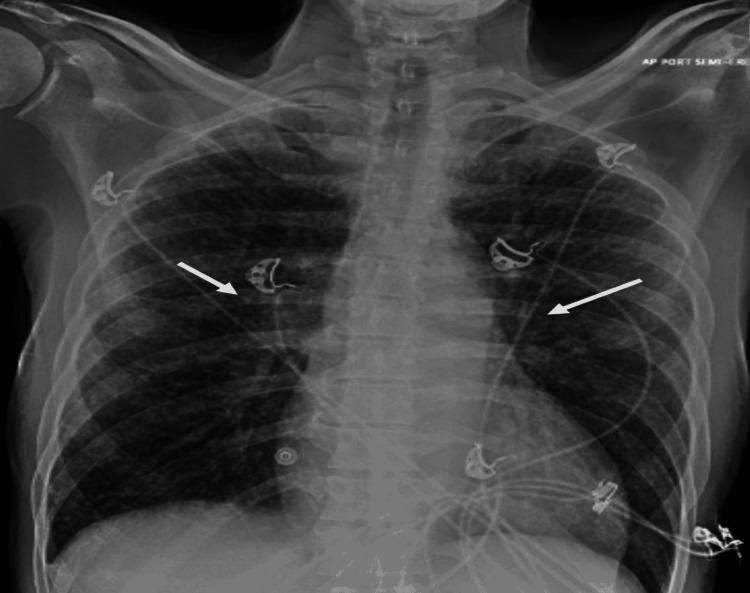
Chest X-ray (anteroposterior view) on presentation. White arrows show bilateral widespread fine reticulonodular opacities.

A 58-year-old male with a past medical history of hyperlipidemia, hypertension, chronic kidney disease (stage 3), and systolic heart failure with reduced ejection fraction, with no significant family history, presented with shortness of breath, productive cough, fatigue, and difficulty in swallowing, along with weight loss of 20-30 pounds over three months. He was a non-smoker and an occasional drinker. He also reported a change in his voice along with constipation over the course of two to three months. On physical examination, the patient was well oriented to time, place, and person and not in apparent distress. No nasal congestion, rhinorrhea, oral pharyngeal exudate, or erythema was present. Pupils were equal, round, and reactive to light. The patient was not in any respiratory distress, and bilateral equal air entry with rhonchi was present. On cardiovascular examination, bradycardia was present (the patient had a prior history of asymptomatic bradycardia with first-degree heart block) with a normal rhythm. The abdomen was soft and flat with no distention, and no palpable mass guarding or rigidity was noted. No focal neurological deficit was present on neurological evaluation. Initial lab work showed leukopenia with a WBC count of 2900 K/UL, hemoglobin of 12.8 g/dL, platelet count of 139,000 per microliter, calcium of 13.7 mg/dL, blood urea nitrogen (BUN) of 42 mg/dL, and creatinine of 2.7 mg/dL (baseline: 1.2-1.4). Angiotensin-converting enzyme level was also done because of hypercalcemia, which was 187 nmol/ml/min. Radio imaging including chest X-ray showed bilateral widespread fine reticulonodular opacities (Figure 1).

**Figure 2:**
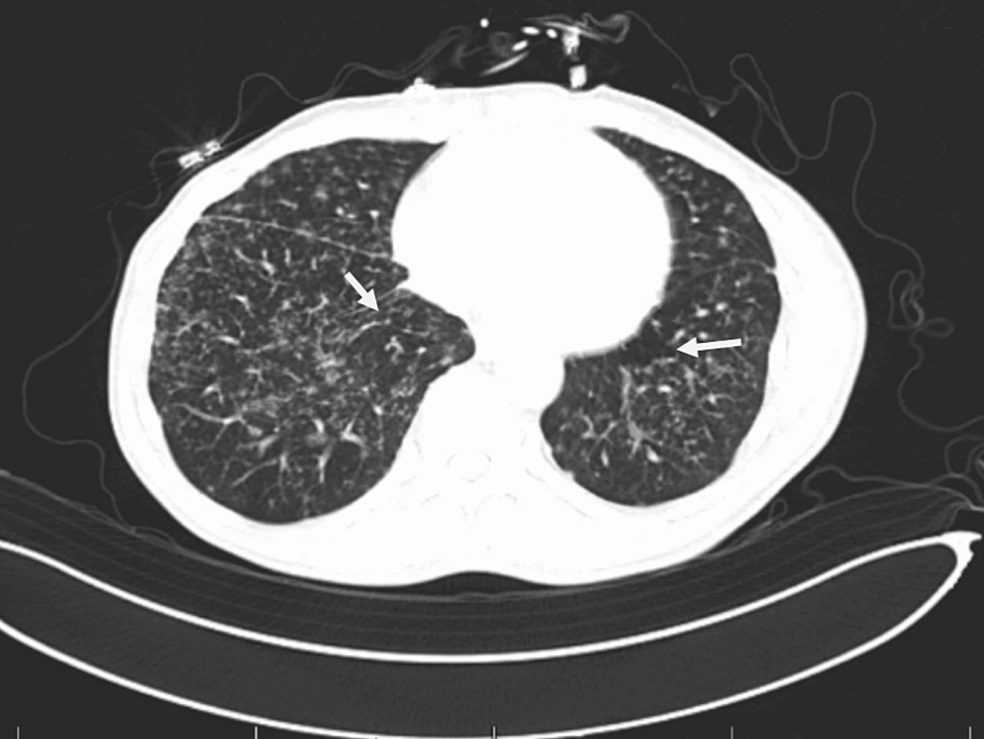
CT of the chest on presentation. White arrows show extensive bilateral reticulonodular opacities throughout the lung parenchyma.

CT scan of the soft tissue neck showed extensive reticulonodular opacities in the upper lung zones bilaterally. CT of the chest (Figure 2) showed extensive bilateral reticulonodular opacities noted throughout the lung parenchyma.

**Figure 3:**
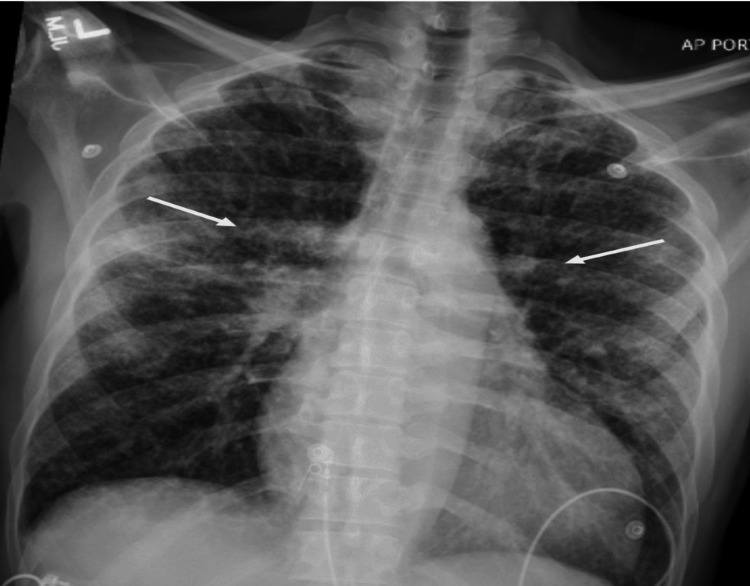
Chest X-ray (anteroposterior view) on day 10 of presentation. White arrows show worsening of bilateral hilar opacities.

On re-evaluation and physical examination, multiple mildly enlarged axillary lymph nodes measuring up to 1.5 cm on the left and 1.2 cm on the right were found. Fine-needle aspiration of the right axillary lymph node was done, which showed blood and cellular debris with rare lymphocytes. Concurrent flow cytometry showed no evidence of a clonal or immunophenotypically abnormal lymphocyte population. Similarly, bronchoalveolar lavage was done, which showed 74% pulmonary macrophages, 17% lymphocytes, neutrophils, a few eosinophils, red blood cells, and very few bronchial cells. Fine-needle aspiration cytology (FNAC) of the right lung was done, which showed noncaseating granulomas. No fungal or acid-fast organisms were identified, and no evidence of malignancy was noted on histopathological evaluation. He also had negative QuantiFERON and negative acid-fast bacilli (AFB) sputum/*Mycobacterium tuberculosis* polymerase chain reaction. For dysphagia evaluation, esophagogastroduodenoscopy (EGD) and a gastric biopsy were done, which showed esophageal stratified squamous epithelium containing acute and chronic inflammation with ulceration and marked reactive atypia, with adjacent glandular epithelium containing acute and chronic inflammation, and no intestinal metaplasia, dysplasia, or malignancy were identified. A transthoracic echocardiogram (TTE) was done, which showed concentric hypertrophy along with normal wall motion and ejection fraction of 50-55% and grade I diastolic dysfunction. Repeat chest X-ray (Figure 3) showed worsening of bilateral hilar opacities.

**Figure 4:**
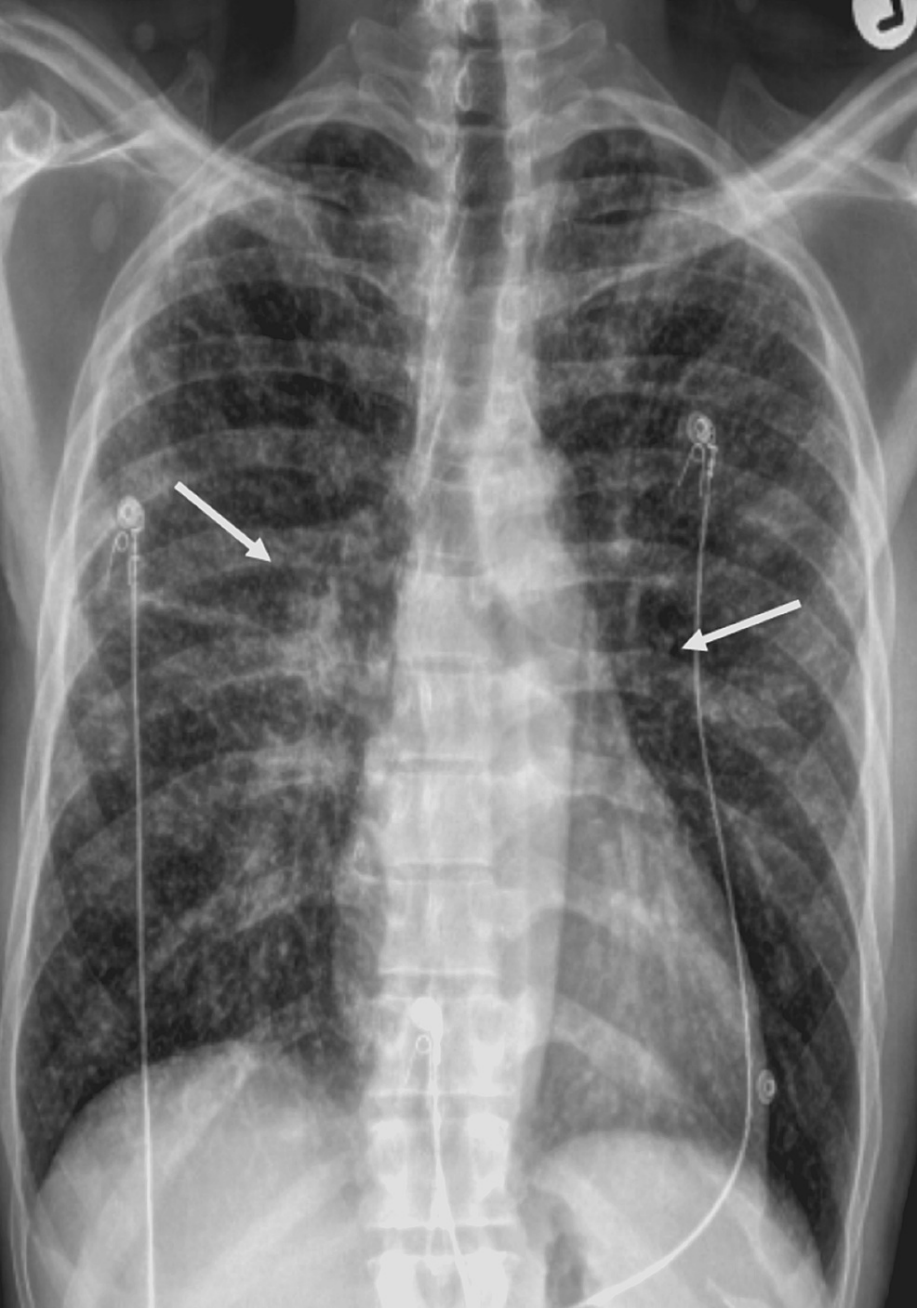
Chest X-ray (anteroposterior view) on day 12 of presentation (two days after starting prednisone therapy). White arrows show significant improvement of the bilateral hilar area.

The patient was started on intravenous fluids and calcitonin, which significantly improved the calcium status of the body as well as improved renal function. Prednisone 40 mg daily was started, which improved his swallowing as well as other symptoms within a couple of days of starting the therapy. A repeat chest X-ray on day 12 showed significant improvement (Figure 4).

The patient was discharged on prednisone 20 mg daily. On his last assessment four months after the initial presentation, his symptoms had significantly improved with adequate weight gain, improvement in swallowing, decreased fatigability, and no respiratory symptoms.

## Discussion

Sarcoidosis is commonly diagnosed clinically along with a histopathological picture of noncaseating granulomatous condition after ruling out other common causes of granulomatous disease such as tuberculosis [3]. Lab workups include complete blood count, liver function test, and renal function test. Since the most frequently involved organ is the lung, the initial set of chest radiographs is advisable. Serum angiotensin-converting enzyme levels can be done, but it lacks both sensitivity and specificity [4]. A large number of patients with sarcoidosis are incidentally diagnosed and a wide variety of symptoms can be involved concomitantly (Table 1). The incidence of hypercalcemia is noted as 11%, while hypercalciuria is more common [1].

**Table 1:** Access trial, organs involved in sarcoidosis.

Organs involved	Number	Percent
Lungs	699	95
Skin	117	15.9
Lymph node	112	15.2
Eye	87	11.8
Liver	85	11.5
Erythema nodosum	61	8.3
Spleen	49	6.7
Neurologic	34	4.6
Parotid/salivary	29	3.9
Bone marrow	29	3.9
Calcium	27	3.7
ENT	22	3
Cardiac	17	2.3
Renal	5	0.7
Bone/joint	4	0.5
Muscle	3	0.4

Dysphagia has been reported to be the most common symptom in patients with esophageal sarcoidosis and can be attributed to various mechanisms such as direct esophageal wall infiltration, extrinsic compression, cranial neuropathy, and brainstem involvement [1]. Other clinical manifestations among patients include weight loss, abdominal pain, odynophagia, hoarseness of voice/dysphonia, and anemia (Table 2) [5].

**Table 2:** Common manifestations in esophageal sarcoidosis.

Most common presentation	Percentage
Dysphagia	91.3
Odynophagia	4.3
Weight loss	21.7
Anemia	4.3
Abdominal/chest pain	8.7
Hoarseness of voice	8.7

The lower esophagus is the most commonly involved (56.5%). The esophageal involvement in sarcoidosis can be classified into four types depending upon the level of involvement and the layer of involvement: superficial mucosal involvement, involvement of the esophageal musculature, direct myenteric involvement, and extrinsic compression [2,5]. Upper GI endoscopy along with histopathological evaluation of the biopsy showing granulomatous infiltration is the key to the diagnosis of esophageal sarcoidosis, and negative special stains for mycobacteria and fungi are required to establish the diagnosis, in addition to evidence of systemic sarcoidosis [3,4]. The management of esophageal sarcoidosis depends upon the presentation. Initially, the patient is started on corticosteroid therapy. The usual starting dosage is prednisone 0.5 mg/kg (30-40 mg daily), and dosage adjustment depends upon the response. The usual duration is uncertain but it is usually 6-12 months. Patients with compressive dysphagia or patients who do not respond well to corticosteroid therapy usually require endoscopic dilatation or esophageal stenting to relieve the symptoms [4].

Renal involvement in sarcoidosis is rare but can be severe by progressing to irreversible end-stage renal failure. The most common presentation is hypercalcemia and hypercalciuria. However, 10% of the patients may also present with nephrolithiasis. Nephrocalcinosis occurs as a result of chronic hypercalciuria and is present in less than 5% of patients with sarcoidosis. It is most often the result of disorders of calcium metabolism inducing calcium renal deposits. The parenchymal involvement is frequently tubulointerstitial nephritis [6]. The most common renal lesion seen on biopsy is granulomatous interstitial nephritis [7]. The incidence and prevalence of these lesions are uncertain; however, it was seen in almost 23% of the patient's in a small case series [8] and 48% in another case series, and most of them were clinically silent [9]. Urinalysis is usually normal; however, sterile pyuria, microscopic hematuria, glycosuria, and hypercalciuria can also be seen. The absence of characteristic kidney biopsy findings does not exclude the diagnosis of renal sarcoidosis, so an effort is put to diagnose the patient clinically and histopathologically from wherever it is accessible and treatment is to be started as early as possible to prevent significant chronic kidney disease [10-12]. Acute symptomatic hypercalcemia is treated with intravenous normal saline infusion. Loop diuretics help in urinary calcium excretion via the thick segment of the loop of Henle. Calcitonin is rarely used for acute hypercalcemia because of its short-acting effect. Glucocorticoids are the mainstay for the long-term treatment of hypercalcemia related to sarcoidosis [13]. The starting dose is prednisone 0.3-0.5 mg/kg/day and the maintenance dose is 5-10 mg/day. The total duration of therapy is 12 months [8]. Chloroquine (200-400 mg daily) or ketoconazole (200-800 mg daily) can be used as an alternative if glucocorticoids cannot be used. Similarly, there are few options such as steroid-sparing agents. The most commonly used medication is azathioprine at a daily dose of 2 mg/kg body weight. However, because of its delayed effect, it needs bridging for the first month of the treatment [14]. Methotrexate is still a second-line steroid-sparing agent with an initial dose of 10-20 mg per week orally or intramuscularly [15]. There has been very limited data regarding the use of mycophenolate mofetil (500-3000 mg/day) as another steroid-sparing agent. ESRD secondary to sarcoidosis is very uncommon; however, young patients with early onset of sarcoidosis can benefit from renal transplantation. There is a very high rate of renal recurrence (17%) after transplantation. Most of the relapses after transplantation are treated with infliximab [15].

Hematological abnormalities associated with sarcoidosis include leukopenia, anemia, and thrombocytopenia [16]. The mechanism is still unknown; however, thrombocytopenia in patients with sarcoidosis has been described mainly based on three mechanisms. The first mechanism is hypersplenism/splenomegaly, which has been seen in almost 6074 cases of sarcoidosis in around 29 publications [17]. The most common mechanism is sequestration in the spleen leading to platelet destruction. Other mechanisms described have been hypersplenism secondary to portal hypertension and peripheral pancytopenia. The second mechanism proposed is bone marrow involvement. In the excess research group, 3.9% of the patients presented with granulomas in the bone marrow [18]. The third mechanism is an autoimmune process, the possible helper T-cells mediated granulomatous response and humoral immune reaction [18,19]. There is not a consensus regarding the treatment modality for thrombocytopenia associated with sarcoidosis; however, the use of steroids has been suggested as a first-line agent with good response and outcome. In patients with emergent situations, methylprednisolone 1 g/day for two days or/and intravenous immunoglobulin (IVIG) 1 g/kg body weight for three days along with platelet transfusions have been recommended. Similarly, steroid-sparing agents such as azathioprine, rituximab, cyclophosphamide, vincristine, and adalimumab can also be used. Splenectomy should be reserved for patients not controlled with steroid, IVIG, or steroid-sparing therapy [19].

## Conclusions

Dysphagia has been reported to be the most common symptom in patients with esophageal sarcoidosis and can be attributed to various mechanisms such as direct esophageal wall infiltration and extrinsic compression. The diagnosis is with biopsy of the most accessible area, which shows noncaseating granuloma. FNAC of the right lung in our patient also showed noncaseating granuloma. Some patients may present with renal involvement and the most common presentation is hypercalcemia with or without hypercalciuria. The initial management is intravenous fluid supplementation to reduce the calcium level of the body. Calcitonin can be used occasionally as well. Intravenous fluid along with loop diuretics significantly improves the renal and calcium status of the body. Some patients with sarcoidosis may also present with hematological disorders, such as leukopenia, anemia, and thrombocytopenia, which significantly respond to the initiation of steroid therapy.
